# Production of high-energy Li-ion batteries comprising silicon-containing anodes and insertion-type cathodes

**DOI:** 10.1038/s41467-021-25334-8

**Published:** 2021-09-15

**Authors:** Gebrekidan Gebresilassie Eshetu, Heng Zhang, Xabier Judez, Henry Adenusi, Michel Armand, Stefano Passerini, Egbert Figgemeier

**Affiliations:** 1 Institute of Power Electronics and Electric Drives, ISEA, RWTH Aachen, Aachen, Germany; 2 Department of Material Science and Engineering, Mekelle Institute of Technology—Mekelle University, Tigray, Ethiopia; 3 Key Laboratory of Material Chemistry for Energy Conversion and Storage (Ministry of Education), School of Chemistry and Chemical Engineering, Huazhong University of Science and Technology, Wuhan, China; 4 Centre for Cooperative Research on Alternative Energies (CIC energiGUNE), Basque Research and Technology Alliance (BRTA), Vitoria-Gasteiz, Spain; 5 Karlsruhe Institute of Technology (KIT), Karlsruhe, Germany; 6 Helmholtz Institute Ulm (HIU), Ulm, Germany; 7 Hong Kong Quantum AI Lab (HKQAI), New Territories, Hong Kong China; 8 Department of Chemistry University of Rome “La Sapienza”, Rome, Italy; 9 Helmholtz Institute Münster (HI MS), IEK-12, Forschungszentrum Jülich, Münster, Germany

**Keywords:** Batteries, Energy, Batteries, Batteries

## Abstract

Rechargeable Li-based battery technologies utilising silicon, silicon-based, and Si-derivative anodes coupled with high-capacity/high-voltage insertion-type cathodes have reaped significant interest from both academic and industrial sectors. This stems from their practically achievable energy density, offering a new avenue towards the mass-market adoption of electric vehicles and renewable energy sources. Nevertheless, such high-energy systems are limited by their complex chemistry and intrinsic drawbacks. From this perspective, we present the progress, current status, prevailing challenges and mitigating strategies of Li-based battery systems comprising silicon-containing anodes and insertion-type cathodes. This is accompanied by an assessment of their potential to meet the targets for evolving volume- and weight-sensitive applications such as electro-mobility.

## Abstract (web summary)

Large-scale manufacturing of high-energy Li-ion cells is of paramount importance for developing efficient rechargeable battery systems. Here, the authors report in-depth discussions and evaluations on the use of silicon-containing anodes together with insertion-based cathodes.

## Introduction

Incentivised by the ever-increasing markets for electro-mobility and the efficient deployment of renewable energy sources, there is a large demand for high-energy electrochemical energy storage devices^1–7^. Lithium-ion batteries (LIBs) utilising graphite (Gr) as the anode and lithium cobalt oxide (LiCoO_2_, LCO) as the cathode have subjugated the battery market since their commercialisation by Sony in the 1990s^8,9^. They are responsible for 63% of worldwide battery sales with an estimated global market value of US$ 213.5 billion by 2020^10^. Within this share, the automotive end-use industry is predicted to lead the overall mass market due to an increasing interest in EVs (http://www.ev-volumes.com/)^11^.

However, despite the striking growth in sales of LIBs worldwide, the practical specific energy of contemporary commercial LIBs (~250 Wh kg^−1^ based on a Gr||lithium nickel manganese cobalt oxide (NMC) cell) is not adequate to achieve the stringent requirements of next-generation batteries^6^. This implies that to stimulate EV market penetration, improvements, mainly including specific energy and energy density (>400 Wh kg^−1^ and > 800 Wh L ^−1^) to enable long-range driving (>500 km), rate capability (i.e. high current density, ≥2 C, the symbol ‘C’ represents the current rate used for cycling rechargeable batteries, *x*C refers to the current required to fully charge/discharge the battery in 1/*x* h), and cost (<125 US$ kWh^−1^, expected in 2022) at the cell level, are urgently required^12,13^.

Transitioning beyond the horizon of prevailing LIBs to avoid ‘driving range anxiety’ and thereby contending with traditional combustion engine vehicles in terms of driving range per charge demands the exploration of novel chemistries and materials. Since the energy density of batteries is determined by Coulombic capacity and cell voltage, the combination of a wide redox-potential gap and high-capacity electrode materials is of fundamental importance. Rechargeable lithium metal (Li^0^)-based batteries (LMBs) have emerged as promising technologies, yet their large-scale deployment has never been feasible except for Li-metal polymer batteries commercialised on a relatively small scale by Bollore (https://www.blue-solutions.com/en/). This is due to the thermodynamic and kinetic instability of the Li^0^/electrolyte interphase induced by dendrite/mossy formation during the plating/stripping processes.

**Fig. 1:**
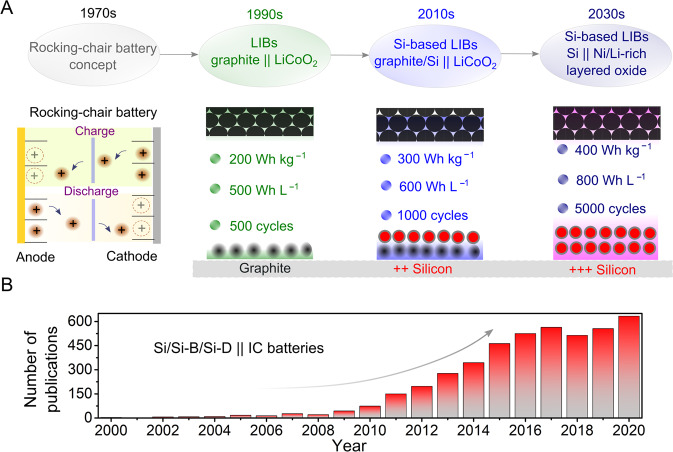
Overview of the development of LIBs over the years. **A** Evolution of LIBs from the rocking-chair battery concept to today’s LIBs and next-generation Si/Si-B/Si-D||IC batteries. Key indicators (specific energy, energy density and cycle life) are comparatively displayed. **B** Research trend in the field of Si/Si-B/Si-D||IC cells. (Last updated on June 28th, 2021 at 2 pm, Berlin time). The key search words used in Scopus^®^ were ‘lithium-ion battery + silicon anode/Si-based/derivatives + insertion and/or intercalation cathode’.

To bypass the challenges associated with Li^0^ anodes, various research and development (R&D) ventures have led to several (high capacity) alternative strategies to replace dendritic Li^0^ deposits while maintaining similar operating voltages and safer electrode materials^5–7^. Accordingly, as illustrated in Fig. 1, silicon (Si), silicon-based (Si-B, e.g. silicon-graphite (Si/Gr)), and silicon derivatives (Si-D, e.g. silicon oxide (SiO_*x*_), silicon oxide-graphite (SiO_*x*_/Gr), silicon nitride (SiN_*x*_), etc.) have been developed as one of the most propitious anode materials due to their various benefits in comparison to other commercial anode materials (Supporting Table 1)^14^. On the other hand, to achieve LIBs with high-energy, high-capacity/high-voltage positive electrodes are a prerequisite. Insertion cathodes (ICs), especially Ni-rich NMC, Ni, Co and Al (dubbed NCA), Li-rich NMC and high-voltage materials, are among the most appealing materials, considering their straightforward chemistries^15^. Thus, rechargeable batteries built by pairing high-capacity, low-potential Si and/or Si-B/Si-D anodes with IC materials have been highly investigated to enable next-generation LIBs. This is evidenced by the increasing number of research works published in the last 5 years (Fig. 1).

From this perspective, we present an in-depth analysis of rechargeable batteries built from Si/Si-B/Si-D anodes coupled with IC cathode materials. In an attempt to avoid simply narrating, ‘who did what, and when?’, the available literature has been critically inspected, and prospects for development opportunities, insight into future research directions and guidelines for the advancement of Si/Si-B/Si-D||IC cells are also given. Furthermore, a detailed analysis of materials chemistry with a focus on evaluating key metrics such as electrochemical performance, economic viability, safety, and reliability is provided.

## Cell chemistry

### Negative electrode chemistry: from pure silicon to silicon-based and silicon-derivative

#### Pure Si

**Fig. 2:**
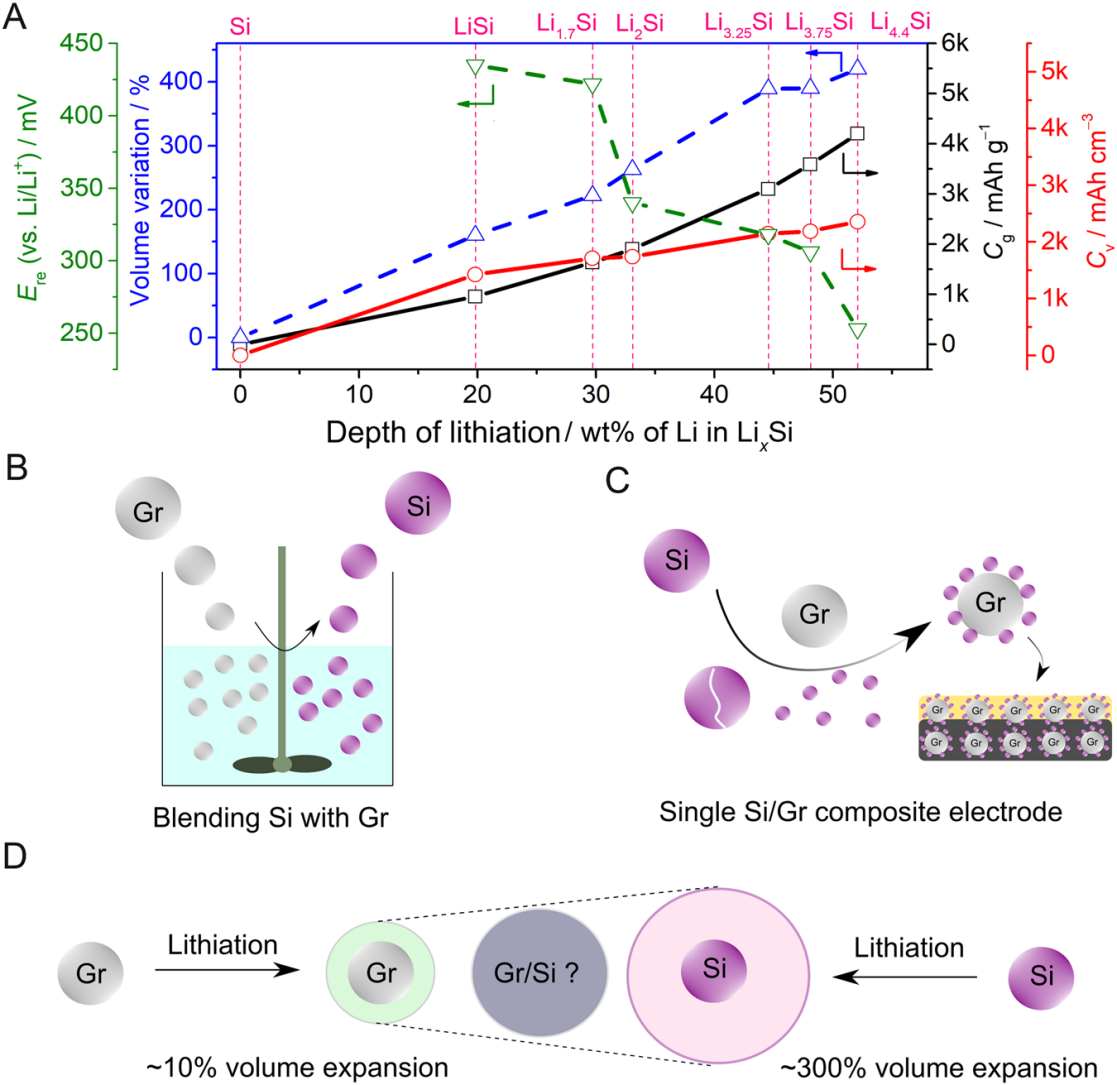
Negative electrode chemistry for pure silicon and Si-based materials. **A** Theoretical capacity [specific (*C*_g_) and volumetric capacity (*C*_v_)], volume variation upon (de)alloying, and reaction potential (*E*_re_) for various lithiated Si electrodes. **B** Mechanical blending of Si with Gr during the electrode fabrication process. **C** Building a single Si/Gr composite electrode. **D** Volume expansion in Si-Gr blend/composite electrodes.

The electrochemical reaction between Li^0^ and elemental Si has been known since approximately the 1970s; in particular, Li–Si alloys (Li_*x*_Si, 0 < *x* ≤ 4.4) were of great curiosity for use as anodes in elevated-temperature molten salt electrolyte batteries operating in the 350–500 °C range^16^. The Li–Si alloy reaction plays a critical role in utilising Si as an anode material. Based on the equilibrium of the Li–Si binary phase diagram, various intermetallic states are favourably formed at distinct thermodynamic voltage plateaus and temperatures (Fig. 2A). The reaction potential decreases with an increasing degree of lithiation (insertion of Li into the active host material). By virtue of its overwhelming attributes, Si has attracted increased attention from the academic and industrial research communities along with policymakers as a next-generation anode material^11^. Nevertheless, the practical application of pure Si- and/or high Si-containing anodes is currently impeded by the presence of multiple inter-related challenges: the enormous volume change (>280%) of Si upon full lithiation induces severe cracking and pulverisation of anodes (at both the active material and electrode levels), a much lower electronic conductivity (*σ*_e_ < 10^−5^ S cm^−1^) and Li^+^ diffusion rate (*D*_Li_^+^, 10^−14^–10^−13^ cm^2^ s^−1^) in high-purity Si compared to that of carbon and graphite (*σ*_e−_, 10–10^4^ S cm^−1^; *D*_Li_^+^, 10^−9^ cm^2^ s^−1^)^17–19^, unstable/dynamic solid electrolyte interphase (SEI) formation, electrode swelling and electrolyte drying^14^.

### Si-based materials: Si-graphite blend/composite, Si-containing functional second phase

To mitigate the challenges linked to pure Si, coupling Si and Gr has been hailed as the most effective strategy towards the commercialisation of Si anode-based high-energy LIBs^17–19^. Adding Gr to Si materials buffers the volume change of the overall composite electrode (not individual Si particles) at a relatively low Si content (~20% Si) because the major portion of the reactive sites for SEI formation is provided by the graphite particles^20^. The addition of Gr to Si also increases the diffusivity (*δ*_e−_ and *D*_Li_^+^) of the electrode and improves its processability in terms of electrode manufacturing (e.g. calendaring), thus enabling high specific, areal and volumetric capacities. Co-utilisation can be achieved by blending Si with Gr during the electrode fabrication process (Fig. 2B) or by building a single Si-Gr composite electrode (Fig. 2C). Moreover, the co-utilisation of Si and Gr can be considered as drop-in technology of graphite, i.e. adopting existing scalable synthesis protocols, establishing commercial production lines, translating into high manufacturability and providing a marginal investment of present graphite-based LIB technology. This accelerates the large-scale deployment of Si-Gr anodes without disrupting established manufacturing lines^21^. To date, battery manufacturers have introduced small amounts of Si (<6–8 wt.%), yet incorporating up to 50–60 wt.% Si remains a serious obstacle due to large volume change-induced SEI failure, accelerated electrolyte decomposition and drying out^14^. The use of a high Si content can only be realised by the synergistic optimisation of various parameters, including polymeric binders compatible with Gr and Si chemistries, functional additives, pre-lithiation, surface functionalisation, protective coatings, etc^20^. The individual materials (i.e. Gr, Si) and their physical blend/composites possess different characteristics and failure mechanisms (e.g. different rates for volume expansion, as shown in Fig. 2D), calling for different diagnostic approaches. Moreover, the difference in the (de)lithiation potentials of Gr and Si (e.g. ~20 mV (LiC_6_) vs. 250 mV (Li_4.4_Si) vs. Li/Li^+^, Fig. 2A) incurs further complexity towards understanding the actual working mechanism of the blended and/or composite electrode systems.

In addition to adding Gr to Si, Si-containing functional second phases, including embedding Si particles into a continuous carbon matrix such as graphene, carbon nanotubes (CNTs), and carbon nanofibres (CNFs), as well as designing Si-containing encapsulated structures, including core-shell, yolk-shell and tailored porous structures, are among the most investigated anode materials at the laboratory scale; however, transfer to the industrial level remains challenging^22^.

### Silicon-derivative materials: SiO_*x*_, SiO_*x*_-Gr and SiN_*x*_

#### SiO_*x*_

Recently, silicon oxide (SiO_*x*_, 0 < *x* ≤ 2) has been investigated as a promising replacement for elemental Si due to its easy synthesis, mild theoretical volume expansion (~118% for SiO compared to Si, ≥300%), and low cost^23^. The blending of Si-rich SiO_*x*_ (*x* ≤ 1) with Gr has become a hotspot leading to its commercialisation in LIBs (usually SiO_*x*_ < 10 wt.%)^7,24^. The SiO_*x*_ family includes silicon monoxide (SiO)-, silicon dioxide (SiO_2_), non-stoichiometric SiO_*x*_ and silicon oxycarbide (Si–O–C)-based anode materials. The O content affects the specific capacity, cycle life and voltage hysteresis (voltage hysteresis refers to the voltage difference between the charge and discharge profiles)^25^. For instance, high O contents result in extended cycle life and high-voltage hysteresis due to the release in the strain and stress of the Li-ion insertion/extraction processes^26^, while lowering the initial Coulombic efficiency (ICE) and rate capability. This is mostly due to the irreversible consumption of active Li^+^ ions (e.g. forming Li_2_O and Li_4_SiO_4_). Although still under investigation, it is generally accepted that the first lithiation of SiO_*x*_ families, for instance, SiO, results in the formation of Li_*x*_Si, Li_2_O (${{{{{\rm{SiO}}}}}}+2{{{{{{\rm{Li}}}}}}}^{+}+2{{{{{{\rm{e}}}}}}}^{-}\to {{{{{\rm{Si}}}}}}+{{{{{{\rm{Li}}}}}}}_{2}{{{{{\rm{O}}}}}}$), Li-silicates (e.g. Li_4_SiO_4_: $4{{{{{\rm{SiO}}}}}}+4{{{{{{\rm{Li}}}}}}}^{+}+4{{{{{{\rm{e}}}}}}}^{-}\to 3{{{{{\rm{Si}}}}}}+{{{{{{\rm{Li}}}}}}}_{4}{{{{{\rm{Si}}}}}}{{{{{{\rm{O}}}}}}}_{4}$; Li_2_Si_2_O_5_: $5{{{{{\rm{SiO}}}}}}+2{{{{{{\rm{Li}}}}}}}^{+}+2{{{{{{\rm{e}}}}}}}^{-}\to 3{{{{{\rm{Si}}}}}}+{{{{{{\rm{Li}}}}}}}_{2}{{{{{{\rm{Si}}}}}}}_{2}{{{{{{\rm{O}}}}}}}_{5}$; Li_6_Si_2_O_7_: $7{{{{{\rm{SiO}}}}}}+{6{{{{{\rm{Li}}}}}}}^{+}+{6{{{{{\rm{e}}}}}}}^{-}\to 5{{{{{\rm{Si}}}}}}+{{{{{{\rm{Li}}}}}}}_{6}{{{{{{\rm{Si}}}}}}}_{2}{{{{{{\rm{O}}}}}}}_{7}$), and Li_2_SiO_3_ (e.g. $3{{{{{\rm{SiO}}}}}}+2{{{{{{\rm{Li}}}}}}}^{+}+{2{{{{{\rm{e}}}}}}}^{-}\to 2{{{{{\rm{Si}}}}}}+{{{{{{\rm{Li}}}}}}}_{2}{{{{{\rm{Si}}}}}}{{{{{{\rm{O}}}}}}}_{3}$).

#### SiN_*x*_

Silicon nitride (SiN_*x*_) is another family of Si derivatives that electrochemically undergoes a two-stage reaction, i.e. a conversion reaction leading to the formation of electrochemically stable and Li-ion conductive Li–Si–N compounds (e.g. Li_2_SiN_2_^27^), followed by an alloying reaction, resulting in Si nanoparticles (SiNPs)^28–30^. Stoichiometrically, increasing the nitrogen content in SiN_*x*_ gradually reduces the reversible capacity while increasing the cycling stability and rate capability^29^. The physical distribution of Li silicon nitride products (Li_*x*_SiN_*y*_) after lithiation reactions greatly affects the cycling performance of SiN_*x*_ electrodes^30^. Despite the promising performances obtained in terms of cycling stability and rate capability, SiN_*x*_ anode-based Li-ion batteries are not commercially viable. To implement SiN_*x*_ in practical cells, strategies that use powder-based electrodes and improve its low first-cycle Coulombic efficiency (e.g. use of additives) need to be adopted.

### Positive electrode chemistry

**Fig. 3:**
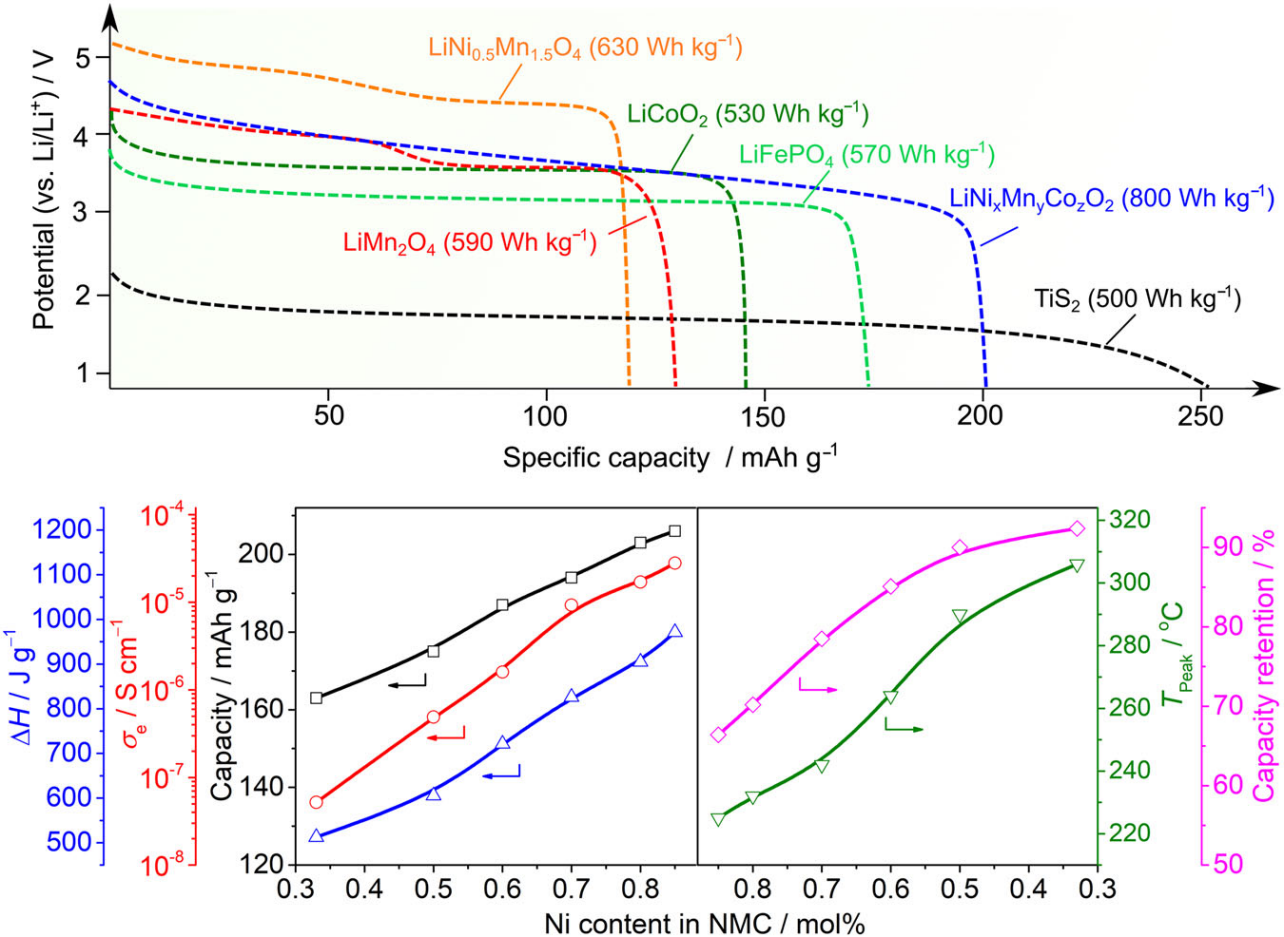
Cell chemistry of typical insertion cathode materials. **Upper plot** Discharge profiles (vs. Li-metal anode) of TiS_2_, LiMn_2_O_4_, LiCoO_2_, LiFePO_4_, LiNi_0.5_Mn_1.5_O_4_ and LiNi_*x*_Mn_*y*_Co_*z*_O_2_. The estimated specific energy of each cathode is shown in parentheses, and the values are obtained by simply considering the working voltage of a Li metal||IC half-cell and the discharge capacity of each cathode. **Lower plot** Effect of the Ni amount on the discharge capacity, thermal stability (peak temperature (*T*_peak_) and heat generation (∆*H*)), capacity retention and electronic conductivity (*σ*_e_) of various NMC materials. Detailed information on these parameters is given in Supporting Table 2.

In contrast to Li-free electroactive materials [e.g. titanium disulfide (TiS_2_)^31^], which require a highly reactive lithium-metal anode^32^, Goodenough and co-workers discovered several important Li-containing cathodes (e.g. LiCoO_2_ (LCO)^33^, lithium manganese oxide (LiMn_2_O_4_, LMO)^34^, and lithium iron phosphate (LiFePO_4_, LFP)^35^) that could not only increase the operational voltage (i.e. from 3 to 4 V) of rechargeable batteries but also permit the production of batteries under less controlled conditions (e.g. a clean room with a relative humidity of ~30%)^9^. In recent years, due to the drawbacks of individual layered cathode materials (e.g. LCO and LMO) and the quest to improve the energy density of LIBs, cathode materials with higher voltage and/or capacity have been proposed, including nickel-rich layered oxides (LiNi_1−*x*_M_*x*_O_2_, M = Co, Mn, and Al), lithium-rich layered oxides (Li_1+*x*_M_1−*x*_O_2_, M = Mn, Ni, Co, etc.), high-voltage spinel oxides (LiNi_0.5_Mn_1.5_O_4_, LNMO), and others, as reported and discussed in the literature^36–38^. Figure 3A comparatively illustrates the discharge profiles of some of the representative cathode materials of each of the families. Considering Li/Li^+^ as a reference redox couple, the cell voltage increases on the order of TiS_2_ (~2.0 V) < LFP (~3.4 V) < LCO ≈ LMO ≈ LiNi_1/3_Mn_1/3_Co_1/3_O_2_ (NMC111) (~3.7 V) < LiNi_0.8_Mn_0.1_Co_0.1_O_2_ (NMC811), ≈LNMO (~4.8 V). Nevertheless, the cell capacity does not follow the same tendency, thus requiring the selection of a particular cell configuration for attaining optimal energies (see the ‘Energy density estimation of full cell’ section).

Ternary oxides such as NMC and NCA can provide theoretical capacities in the range of 200–210 mAh g^−1^ in the voltage window of 3.0–4.3 V vs. Li/Li^+^^39^. The logic behind the mixing of Ni, Co and Mn or Al is attributed to their crucial elemental physical properties, including the high capacity and voltage of Ni, fast charge-discharge kinetics of Co, structural cycling stability of Mn and improved thermal stability of Al^36–38^. Regarding NMC variants, increasing the Ni content from NMC111 to NMC811 and beyond provides highly encouraging features, including improved specific capacity, higher diffusivity (both *σ*_e−_ and *D*_Li+_), and decreased material use and overall cost (Fig. 3B). Due to having both higher electronic conductivity (~10^−8^ S cm^−1^ for NMC111 vs. ~10^−5^ S cm^−1^ for NMC811) and Li^+^ diffusivity (10^−11^–10^−12^ cm^2^ s^−1^ for NMC111 vs. 10^−8^–10^−9^ cm^2^ s^−1^ for NMC811), the rate capability of NMC811 is much higher^36^. However, this comes at the expense of other stringent requirements of EVs, such as thermal stability (i.e. safety) and capacity retention. For example, a higher Ni content in the NMC cathode cannot only boost the specific energy of electric vehicle batteries but also increases the risk of thermal runaway and decreased cycle life (Fig. 3B). Moreover, Ni-rich cathode materials, e.g. NMC811, incur extra processing costs. Their production, handling and storage (due to their high sensitivity to moisture and air) and their integration at the multi-ton scale present more difficulties than their low Ni content counterparts. NCA shares similarities with NMC811, i.e. delivering high specific energy and power. Although the cyclability of NCA-based cells tends to be better, the 10 wt.% Co of NCA-based cells is higher than that in NMC811, making them less cost-effective. Nonetheless, increasing the Ni content of NCA to over 80 wt.% to boost the cell capacity is still an option that is currently being investigated in the industrial battery research field.

## Energy estimation of full cells

**Fig. 4:**
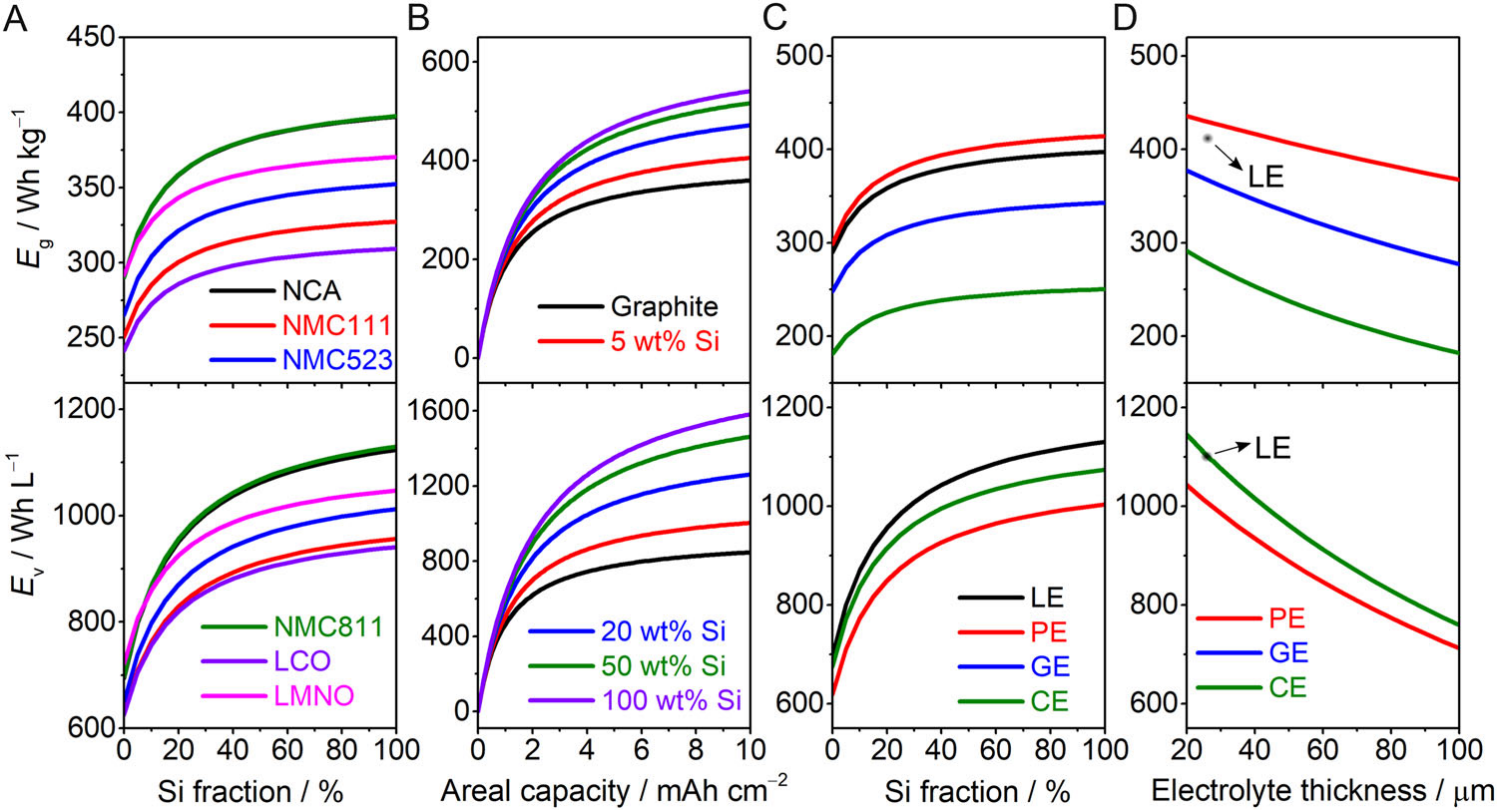
Estimated specific energy (Eg) and energy density (Ev) values of Si/Si-B/Si-D||IC cells at 25 °C. **A** Specific energy and energy density vs. Si fraction for LE-based cells at a fixed areal capacity of 3 mAh cm^−2^. **B** Specific energy and energy density vs. areal capacity for LE-based cells at various Si contents. **C** Specific energy and energy density for Si||NMC811 cells using various kinds of electrolytes at a fixed areal capacity of 3 mAh cm^−2^. **D** Effect of electrolyte thickness on the specific energy and energy density of Si/Gr (20/80, by wt.)||NMC811 cells at a fixed capacity of 5 mAh cm^−2^ (LE liquid electrolyte, PE polymer electrolyte, GE glass electrolyte (LIPON), CE ceramic electrolyte (LLZO)); note that the *E*_v_ values for GE and CE overlap in **C** and **D**. All the calculation details are in Supporting Tables 3 and 4.

The specific energy (*E*_g_) and energy (*E*_v_) density of various cathode materials as a function of the weight fraction of Si at a fixed areal capacity of 3 mAh cm^−2^ in a liquid electrolyte (LE) in Si/Si-B/Si-D ||IC cell configurations are depicted in Fig. 4A. For a fixed fraction of Si (i.e. 60 wt.%), the energies are found in the order NMC811~NCA (388 Wh kg^−1^, 1087/1081 Wh L^−1^) > LMNO (LiMn_1.5_Ni_0.5_O_2_) (364 Wh kg^−1^, 1018 Wh L^−1^) > NMC532 (345 Wh kg^−1^, 977 Wh L^−1^) > NMC111 (321 Wh kg^−1^, 925 Wh L^−1^) > LCO (304 Wh kg^−1^, 911 Wh L^−1^), evidencing the promising prospect of Si (60 wt.%) || NMC811 in terms of both *E*_g_ and *E*_v_. Variation in the Si fraction greatly affects *E*_v_ more than *E*_g_. Moreover, for Si > 60 wt.%, the gain in energy density with an increasing Si fraction significantly diminishes.

For commercial applications (EVs and the aviation industry), battery systems require thick electrodes with high specific capacity (i.e. high areal capacity), as they relatively decrease the fraction of non-electroactive materials (e.g. current collectors and separators)^40,41^. Due to the repetitive large volume change associated with Si anodes and the challenges stemming from ICs (active oxygen release and cation migration), achieving long-term cycling stability at high areal capacities is difficult. For the graphite||NMC811 cell using LE, the improvement in both *E*_g_ and *E*_v_ is noteworthy when the areal loading increases up to ~5 mAh cm^−2^. For areal loadings above 5 mAh cm^−2^, the contribution is less significant (Fig. 4B). However, in the case of Si||NMC811 batteries, the *E*_g_ and *E*_v_ values are very sensitive to the areal capacity. The energy density increases up to an areal capacity of 10 mAh cm^−2,^ and the effect of areal capacity is strong up to 80 wt.% Si, with its influence becoming weaker with higher fractions of Si^42^. For example, increasing the areal capacity from 5 to 10 mAh cm^−2^ brings about a 30% increase in *E*_v_, reaching an extremely high value of ~1600 Wh L^−1^ (Fig. 4B). This highlights the importance of a thick electrode in realising high-energy-density Si/Si-B/Si-D||IC cells (cf. ‘Mitigation strategies’).

The specific energy and energy density of a Si||NMC811 cell in the presence of different electrolytes, i.e. LE, polymer electrolyte (PE), glassy electrolyte (GE), and ceramic electrolyte (CE), at various fractions of Si is given in Fig. 4C for a fixed areal capacity of 3 mAh cm^−2^. Inorganic solid electrolytes (CE and GE) with a much higher density (e.g. 5.1 g cm^−3^ (CE) vs. 1.9 g cm^−3^ (GE) vs. 1.2 g cm^−3^ (PE) and 1.1 g cm^−3^ (LE))^17^ hardly achieve *E*_g_ values higher than 250 Wh kg^−1^ for CE and 343 Wh kg^−1^ for GE. Nevertheless, PE and LE present nearly similar *E*_g_ values, reaching 414 and 398 Wh kg^−1,^ respectively. Regarding *E*_v_, the four electrolytes present similar values (LE (1130 Wh L^−1^), GE~CE (1074 Wh L^−1^), and PE (1004 Wh L^−1^)). This suggests that the density of the electrolyte greatly influences the energy density of Si/Si-B/Si-D||IC cells, particularly *E*_g_.

Further estimations of the energies shown in Fig. 4D reveal that the thickness of the electrolyte greatly affects the energy density of Si/Si-B/Si-D||IC cells, particularly for the CE-based cell. For example, at a fixed electrolyte thickness of 25 µm, all four types of cells show high *E*_v_ (>1000 Wh L^−1^); however, the CE-based cell has the lowest *E*_g_ value of 281 Wh kg^−1^. This implies that ultrathin CE membranes (<25 µm) are vital for achieving respectable *E*_g_ values for CE-based cells.

## Challenges of Si/Si-B/Si-D||IC cells

**Fig. 5:**
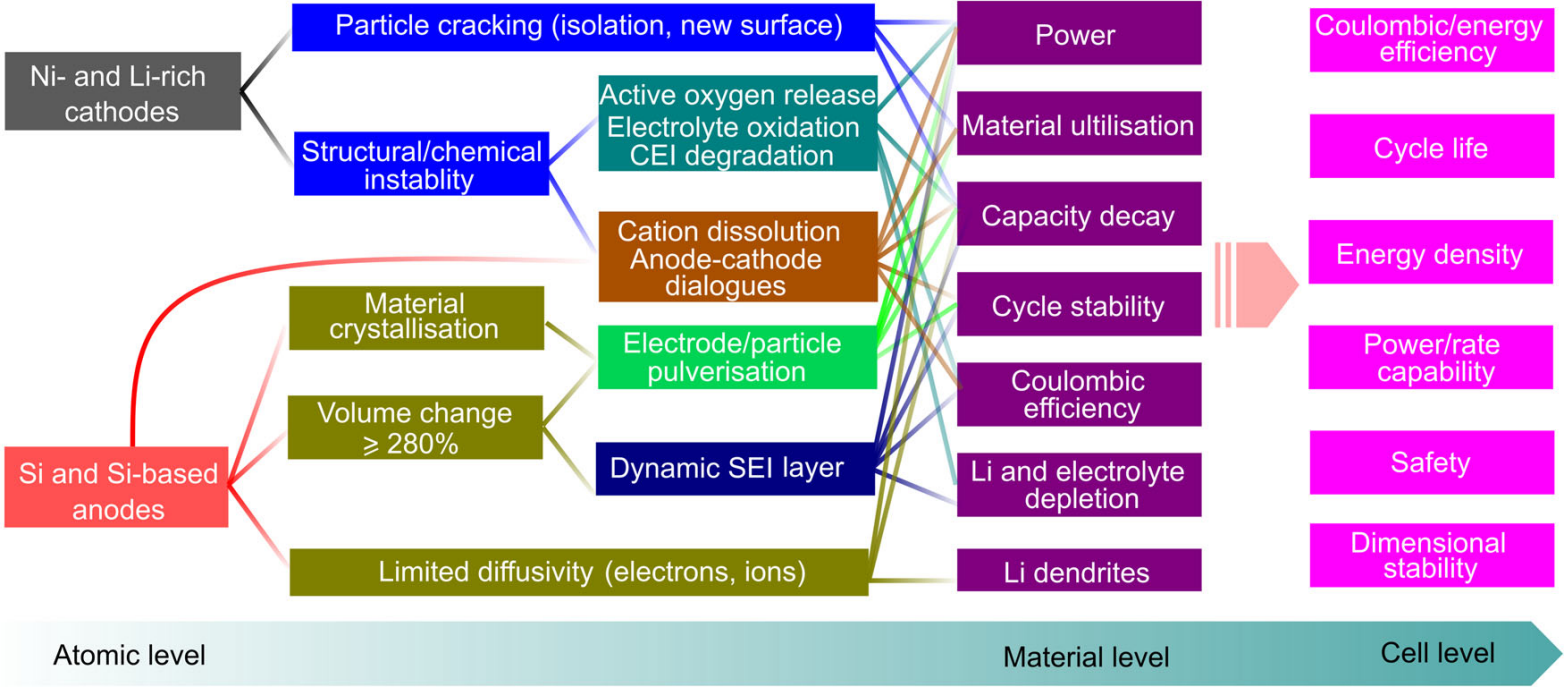
Correlation between the cell-level performance and atomic/material-level properties for high-energy Si/Si-B/Si-D||IC cells. The main indicators for cell-level performance (e.g. Coulombic/energy efficiency, cycle life, energy density, power/rate capability, safety, dimensional stability) are displayed based on the features of the electrode materials (e.g. power, material utilisation, capacity decay, cycling stability, Coulombic efficiency, Li and electrolyte depletion and Li dendrites). Furthermore, the atomic-level properties (e.g. particle cracking, structural/chemical instability, material crystallisation, volume change and limited diffusivity) that are responsible for the fundamental properties at the material level are described.

Towards the use of Si/Si-B/Si-D||IC cells in emerging applications (e.g. EVs and the integration of renewable energy sources), specific energy as well as energy density together with power/rate capability, cycle life, Coulombic/energy efficiency, safety, and cost have to be assessed. These cell-level features are tightly bound to the intrinsic properties of the individual cell components (i.e. anodes, cathodes, binders, conductive additives, electrolytes, etc.) at both the atomic and material levels and their interactions, as illustrated in Fig. 5. While the material-level performances are highly impacted by the atomic-level properties, the atomic-level properties in turn strongly dictate the cell-level performances. Although it is difficult to establish a fine correlation between cell-level performance and atomic-level properties, the intention here is to visualise the possible atomic features that are responsible for the different degradation patterns observed in Si/Si-B/Si-D||IC cells. Note that the system-level design (e.g. shape/size of the cell, 21,700 vs. 18,650 cells^43,44^) is equally important to the individual compartments, but its discussion is beyond the scope of this manuscript.

At the anode side, Si/Si-B/Si-D-based materials undergo large volume changes during (de)lithiation processes, which may cause electrode/particle pulverisation, resulting in a dynamic SEI (which continuously and reversibly evolves with cycling). Consequently, the efficient implementation of electroactive materials is hindered, in particular with Li, when electrolyte depletion is promoted, worsening the rate capability, cycle stability and Coulombic/energy efficiencies. The very limited diffusivity towards electrons and ions of Si/Si-B/Si-D anodes not only results in sluggish kinetics of the redox reaction and capacity decay but also increases the risk of growing Li^0^ deposits due to the rapid and uneven Li nucleation at the active site of the anode upon fast charging^45^. Bi-phasic crystallisation during the electrochemical lithiation of Si (Eqs. 1 and 2 for discharging and Eq. 3 for charging) leads to electrode/particle pulverisation, once again resulting in a lower rate capability, poor material utilisation, accelerated capacity decay, and decreased cycle life.

1 $${{{{{\rm{Si}}}}}}({{{{{\rm{crystalline}}}}}})+x{{{{{{\rm{Li}}}}}}}^{+}+x{{{{{{\rm{e}}}}}}}^{-}\to {{{{{{\rm{Li}}}}}}}_{x}{{{{{\rm{Si}}}}}}({{{{{\rm{amorphous}}}}}})$$

2 $${{{{{\rm{Si}}}}}}({{{{{\rm{crystalline}}}}}})+(3.75-x){{{{{{\rm{Li}}}}}}}^{+}+(3.75-x){{{{{{\rm{e}}}}}}}^{-}\to {{{{{{\rm{Li}}}}}}}_{15}{{{{{{\rm{Si}}}}}}}_{4}({{{{{\rm{crystalline}}}}}})$$

3 $${{{{{{\rm{Li}}}}}}}_{15}{{{{{{\rm{Si}}}}}}}_{4}\left({{{{{\rm{crystalline}}}}}}\right)\to {{{{{\rm{Si}}}}}}\left({{{{{\rm{amorphous}}}}}}\right)+{{{{{\rm{y}}}}}}{{{{{{\rm{Li}}}}}}}^{+}+{{{{{\rm{y}}}}}}{{{{{{\rm{e}}}}}}}^{-}{+{{{{{\rm{Li}}}}}}}_{15}{{{{{{\rm{Si}}}}}}}_{4}({{{{{\rm{residual}}}}}})$$

At the cathode side, IC materials such as Ni- and Li-rich oxide cathodes suffer from structural and chemical instability, leading to active oxygen release, electrolyte oxidation on the cathode surface and cathode electrolyte interphase (CEI) degradation. Such factors could accelerate the depletion of both the Li inventory and the solvent and salt of the electrolyte. This is detrimental to the power, capacity retention and Coulombic/energy efficiencies of Si/Si-B/Si-D||IC cells. In addition, acidic species (e.g. HF, POF_3_ and PF_5_) generated via electrolyte degradation may dissolve the transition metal cations, causing anode–cathode crosstalk, which in turn leads to the catalytic decomposition of the SEI. This also largely determines the key performance indicator parameters at the material level, thus ultimately affecting the performance of Si/Si-B/Si-D||IC cells (Fig. 5). On the other hand, the gases formed by electrolyte degradation and/or active oxygen release may mechanically detach active electrode materials from current collectors, expose new surfaces and even lead to particle cracking of electroactive materials^46^. This is deleterious, especially for the power, material utilisation and capacity retention performance of cells.

Despite the recent surge of publications/reports related to Si/Si-B/Si-D||IC cells, considerable effort has been dedicated to the discovery of new materials and their tests in half-cell setups (Si/Si-B/Si-D||Li and IC||Li cells)^14,17,24,25^. Few works have been done on the design and formulation of Si/Si-B/Si-D||IC full cells, leaving an insurmountable gap for transferring the knowledge and know-how accumulated in laboratories to industrial sectors (a requirement for commercial feasibility)^47–49^. Apart from challenges linked to individual cell components, parameters related to cell design enlisting electrolyte/active material (E/AM, a parameter for quantifying the amount of electrolyte) ratio, negative/positive (N/P ratio, a parameter for quantifying the extra capacity of negative electrodes) capacity ratio and data projection from half-cell to full cell must be analysed.

When evaluating the performance of Si/Si-B/Si-D||IC cells employing flooded electrolyte cell conditions, the Li inventory and N/P ratio is of little significance to improve the technological readiness of new materials discovered in laboratories. Converting the results obtained from half-cells to full cells is not straightforward due to the excessive amounts of Li^0^ used in half-cells. For instance, a Li^0^ anode with a thickness of >50 µm, which is generally employed for evaluating the electrochemical properties of laboratory-scale Si/Si-B/Si-D and IC electrodes^17^, corresponds to a negative electrode capacity that is higher than 10 mAh cm^−2^. When assuming an achievable positive electrode capacity of 3 mAh cm^−2^ for either Si/Si-B/Si-D anodes or Ni-/Li-rich cathode materials, the N/P ratio in the half-cell is always higher than 3.3, which is considerably higher than that in full cells (i.e. N/P ratio = ~1.1). Compared to the full cell, the nearly ‘infinite’ supply of active Li^+^ ions from the metal anode can sufficiently compensate for the irreversible capacity loss during cycling, which may mask the intrinsic properties of novel materials; as a result, difficulties may arise when projecting data from the half-cell to the full cell.

## Mitigation strategies

To evaluate the strategic solutions for the challenges linked to full cells constructed from Si/Si-B/Si-D anodes and ICs, various criteria should be considered for the adequate evaluation of cell performances, namely, energy efficiency, service life (cycle/calendar life), energy density, rate capability, dimensional stability, safety and cost, which are discussed below.

### Redox reaction efficiencies and cycle life

**Fig. 6:**
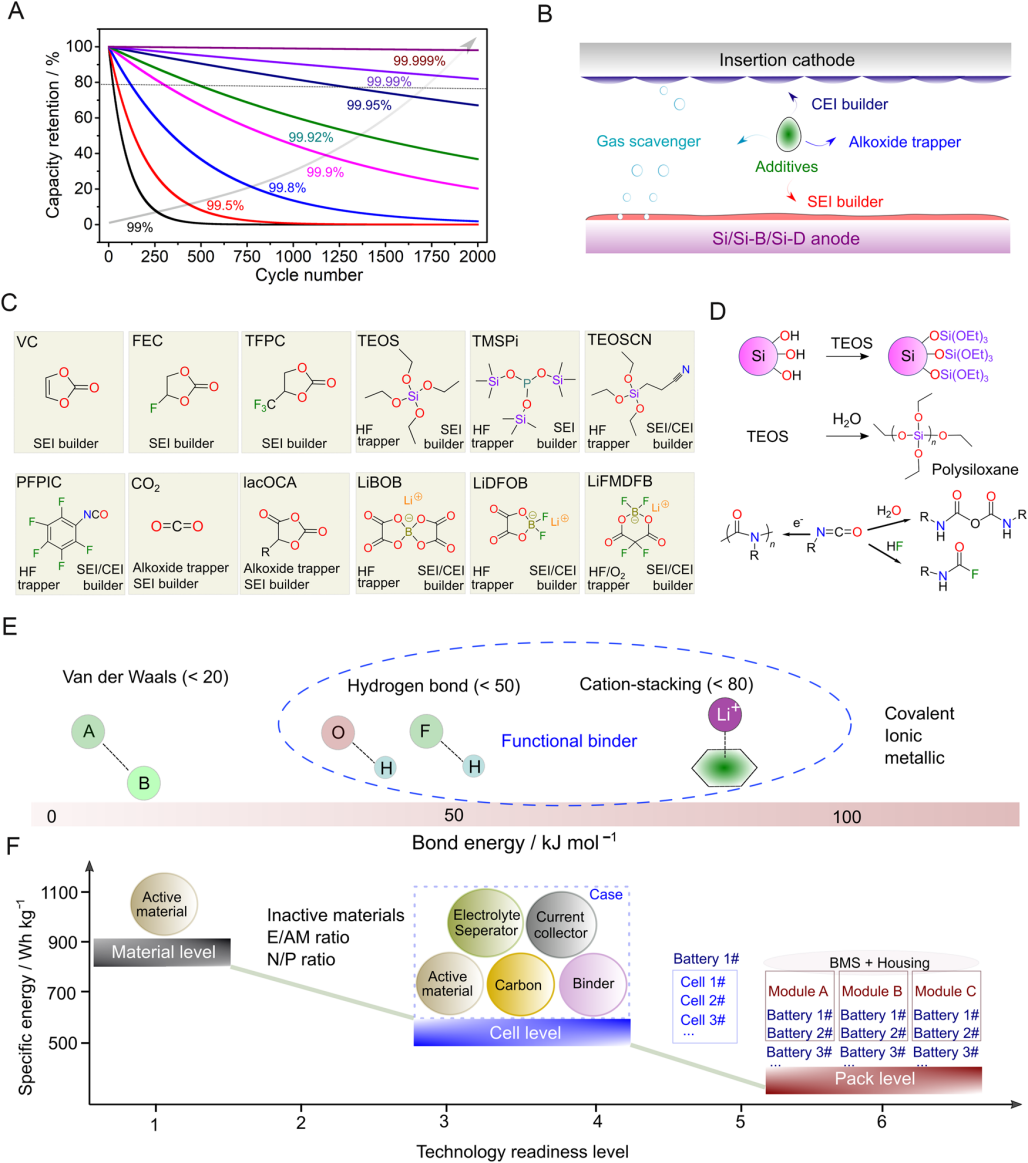
Mitigating strategies for improving the performance of Si/Si-B/Si-D||IC cells. **A** Influence of CE on capacity retention in a hypothetical Si/Si-B/Si-D||IC full cell. **B** Role of an electrolyte additive in Si/Si-B/Si-D||IC cells. **C** Chemical structures and functions of popular electrolyte additives. **D** Chemistry of representative electrolyte additives. **E** Bond energies of various interactions existing in battery materials. **F** Evolution of specific energy from the material to the cell and pack levels.

For practical applications such as EVs and consumer electronics, the Coulombic efficiency (CE) and energy efficiency (EE) are crucial metrics to evaluate the electrochemical energy storage performance of a cell, as they correlate to the lifetime and economy of a battery. CE is the most useful parameter dictating the reversibility of cell processes and service life (i.e. cycle life and calendar life)^47,50,51^. CE is defined by ${{{{{\rm{CE}}}}}}={Q}_{{{{{{\rm{d}}}}}}}/{Q}_{{{{{{\rm{c}}}}}}}\times 100 \% ={\int }_{0}^{t}{I}_{{{{{{\rm{d}}}}}}}{{{{{{\mathrm{d}}}}}}t}/{\int }_{0}^{t}{I}_{{{{{{\mathrm{c}}}}}}}{{{{{{\mathrm{d}}}}}}t}\times 100 \%$, where *Q*_d_ and *Q*_c_, *t*, and *I* are the discharge capacity, charge capacity, time and current, respectively. Figure 6A illustrates the impact of CE on the capacity retention of a theoretical Si/Si-B/Si-D||IC cell, assuming that the CE < 100% is completely related to the irreversible loss of active Li. For example, when the CE is 99%, the remaining available Li after 50 cycles is only 60.5%, i.e. ~40% loss of active Li. After 500 cycles, full Si/Si-B/Si-D||IC cells only deliver 0.66%^49^ of their original capacity. With the state-of-health (SOH, here defined as the ratio between the maximum practical capacity and the theoretical capacity of a battery), which is a threshold set at ~80% after 1000 cycles and is required for the practical use of Si/Si-B/Si-D||IC cells in the electric vehicle industry^11,12,21^, CEs of 99.95% and above are the target. In addition to the irreversible loss of the Li inventory during each cycle, lower CE values also imply the consumption of large amounts of electrolyte, leading to electrolyte drying and an increase in impedance. EE, defined as ${{{{{\rm{EE}}}}}}={\int }_{0}^{{t}_{{{{{{\mathrm{d}}}}}}}}{V}_{{{{{{\rm{d}}}}}}}{I}_{{{{{{\rm{d}}}}}}}{{{{{{\mathrm{d}}}}}}t}/{\int }_{0}^{{t}_{{{{{{\mathrm{c}}}}}}}}{V}_{{{{{{\rm{c}}}}}}}{I}_{{{{{{\rm{c}}}}}}}{{{{{{\mathrm{d}}}}}}t}\times 100 \%$ (where *V*_d_ and *V*_c_ represent the average discharge and charge voltages, respectively), is another crucial parameter of battery materials that generally tends to go unnoticed^50^. The energy efficiency differs from material to material for anodes: graphite (~94%) > soft carbon (93%) > silicon/graphite (~89%); and cathodes: LNMO (~97%) ≥ NMC (96%) ≥ LR-NMC (~85%)^52^. Achieving high CE in Si/Si-B/Si-D||IC cells involves circumventing issues in both the anode and cathode compartments. Owing to the high degree of sensitivity of CE, its improvement affects the cell cycle life and the improvement of EE. The strategies for improving the CE and EE can be summarised by several approaches, including electrolyte development, electrode design, pre-lithiation, polymeric binders and conductive additives. However, only the critical factors are discussed below.

### Electrolyte design

Incorporating a small dose of salt and/or molecular additives (≤10% by wt. or vol.; e.g. vinylene carbonate (VC), fluoroethylene (FEC)) into the electrolyte solution is hailed as the most efficient, economical and scalable strategy to combat the very large volume changes as well as the unstable SEI/CEI layer^14^. To avoid the complexity linked to the use of a mixture of various electrolyte additives usually adopted in commercial LIBs, the design of multifunctional electrolyte constituents is of paramount importance, particularly for Si/Si-B/Si-D||IC cells. The development of additives that possess either multiple functionalities or tailored synergistic effects is required to enable Si/Si-B/Si-D||IC battery systems. In view of the various methods to boost the CE, electrolyte additives can be categorised into different classes (Fig. 6B): (1) surface chemistry (SEI and CEI layers) modifiers, (2) acidic/protonic species scavengers, and (3) reactive species/molecules trappers/transformers.

To enable the practical application of Si/Si-B/Si-D||IC cells, SEI/CEI-forming additives are indispensable to improve their cyclability by suppressing parasitic reactions related to electrode-electrolyte interphases. Such additives possess electrochemically reducible and oxidisable functional moieties^53^. Designer additives used as precursors to build robust SEI/CEI should result in compounds with (1) a high shear modulus, which is essential for boosting the mechanical/chemical stabilities^54^; (2) a low alkalinity/basicity (i.e. low acid-base reactivity), which is critical for suppressing the chemical and electrochemical degradations of electrolytes and electrodes^55^; (3) high Li^+^ transport and negligible diffusion of electrons and electrolyte; and (4) a low solubility in alkyl carbonate solvents, which is beneficial for reducing the reactivity of acidic species and other trace impurities (HF, POF_3_, H_2_O, PF_5_, among others)^56^. In general, electrolyte additives are depleted during each cycle; hence, their early effect (e.g. building the SEI/CEI) should provide sustained benefits throughout the cycling process.

Figure 6C displays a variety of electrolyte additives proven to effectively enhance the CE of Si/Si-B/Si-D||IC full cells. Although widely used as film additives, VC possesses high impedance, thus exhibiting a poor rate capability, as well as serious gas evolution. However, when FEC is used, rapid consumption of the electrolyte additive at elevated temperatures occurs^57,58^. Lewis acid-induced defluorination of FEC results in the formation of reactive species (e.g. HF, HPO_2_F_2_), damaging the SEI/CEI and shortening the lifetime of the battery^59^. Trifluoropropylene carbonate (TFPC) possesses an electron-withdrawing trifluoromethyl group that is capable of improving the cycling stability by regulating the structure and composition of the interphases, outperforming FEC^60^.

Molecular additives containing Si–O moieties such as silanes and their derivatives play multiple roles by forming (–O–Si–O–)_*n*_ polymer-derived passivation layers, scavenging HF among other acidic species due to the strong affinity of HF to Si. –N=C=O-containing additives form a robust SEI/CEI layer and capture trace amounts of HF and water in electrolyte solutions (Fig. 6D)^61^. Nitrile (−C≡N)-functionalized silane (e.g. 2-cyanoethyl triethoxysilane (TEOSCN)) forms highly conductive and mechanically stable SEI/CEI layers, leading to an improvement in performance at elevated temperatures (45 °C, starting temperature for the thermal decomposition of LiPF_6_) compared to the electrolyte without TEOSCN (1 M LiPF_6_ in EC/DEC (1/1, w/w))^54^.

CO_2_, as a precursor to form a highly regulated passivation layer, likely rich in Li_2_CO_3_^62,63^ and Li_2_C_2_O_4_^64^, serves as a reactive-type additive transforming highly soluble and reactive lithium alkoxides (ROLi) into lithium alkyl carbonate (ROCO_2_Li). Thus, the highly nucleophilic ROLi is trapped, thereby deactivating the alkoxide-mediated transesterification of ethyl methyl carbonate (EMC)^14^. To avoid severe safety issues and critical challenges linked to the direct utilisation of solid CO_2_ (dry ice) in pouch cells before battery sealing, the use of in situ (electro)chemically CO_2_ releasing additives (e.g. 5-methyl-1,3-dioxolane-2,4-dione (lacOCA)^65^) has been reported in the literature.

Salt-type/ionic compounds are an alternative group of additives and/or co-salts for Si/Si-B/Si-D||IC cells. Electrolyte salts can form stable SEI/CEI via cation and/or anion functionalities, modifying the surface chemistry as sacrificial additives. This includes salts such as lithium bis(oxalato)borate (LiBOB), lithium difluoro(oxalate)borate (LiDFOB), and lithium fluoromalonato(difluoro)borate (LiFMDFB), either in standalone or in combined form, leading to better Si/Si-B/Si-D||IC battery performances even at higher temperatures (45–85 °C)^66,67^. Imide (e.g. lithium bis(fluorosulfonyl)imide (LiFSI))^68,69^, imidazole-type (e.g. lithium 4,5-dicyano-2-(trifluoromethyl)imidazolide (LiTDI))^70^ and their modified versions are suitable co-salt alternatives that can replace LiPF_6_. Such salt anions, endowed with electrochemically active covalent bonds (e.g. S–F, S–N), act as a source of effective interphase building compounds rich in LiF and Li_3_N; hence, they are potential candidates for Si/Si-B/Si-D||IC cells.

### Pre-lithiation

Pre-lithiation, referring to the incorporation of active Li into the cell (mainly at the negative electrode in the case of Si/Si-B/Si-D||IC cells) before operation, is of particular interest to LIB manufacturers^71^. Presently, the main pre-lithiation routes include ex situ or in situ electrochemical pre-lithiation, chemical pre-lithiation by active reactants, direct contact with metallic Li, pre-lithiation with electrode additives (both on negative and positive electrodes), and overcharged cathodes. For practical applications, the pre-lithiation approach should be simple, scalable, cost-effective, and safe. The pre-lithiation compound(s) should fulfil the following requirements: (1) possess high ‘donor’ lithium-ion capacity during the initial charge/discharge steps with no negative effects on electrochemical performance, such as cycle life and rate capability; (2) offer a controllable pre-lithiation degree, critical for matching the capacity between the cathode and anode; (3) have good compatibility with existing battery fabrication processes, i.e. drop-in technology; and (4) have low cost and high safety^72^. However, most of the reagents proposed thus far require processing in a dry atmosphere, which substantially increases the cost of cell production.

### Design of polymeric binders

The use of custom-built polymeric binders is another appealing approach^73,74^. Due to the huge mechanical stress caused by the large volume change of Si/Si-B/Si-D anodes, the binding actions fail upon cycling, leading to particle pulverisation and uncontrollable growth of the electrode/electrolyte interphases. The harsh conditions (e.g. large volume change associated with anode and cation dissolution and oxygen release on the cathode) linked to the use of Si/Si-B/Si-D||IC cells infer that the knowledge accumulated from state-of-the-art anodes (e.g. graphite and Li_4_Ti_5_O_12_) and cathodes (e.g. LiCoO_2_ and low Ni variants of NMC) may not be useful. Hence, the binder development for Si/Si-B/Si-D||IC needs to be addressed from a new perspective. The adhesive property of polymeric binders is closely related to their chemical nature, associated mode of interactions and nature of active materials as well as carbon conductive additives. For chemistries experiencing very large volume changes, polymeric binders with strong yet reversible interactions are important^14^. This aspect highlights the necessity of balancing the bond strength and reversibility (bond recovery) of the desired binder molecules. Figure 6E depicts the different bonding strengths and comfort zones for the development of potential binders for Si/Si-B/Si-D||IC battery systems. In the development of tailored polymeric binders, reactive functional groups such as −OH, −COOH, and −NH_2_, which are capable of forming strong hydrogen bonds, ion-dipole interactions, and even chemical bonds that are far stronger than van der Waals forces, play a crucial role^73,74^.

### Particle and alloy engineering

The electrochemical performance of Si, Si-B and Si-D anodes is greatly influenced by Si particle size, surface area, surface oxygen content, hydroxyl (−OH) content, and morphology. For instance, the ICE is inversely correlated with surface area and oxygen content. Thus, surface modification/treatment along with alloy formation decreases the irreversible capacity loss and improves the Coulombic and energy efficiencies by mitigating volume expansion and minimising the surface area (https://projects.leitat.org/wp-content/uploads/2016/11/4-3M-Si-alloy-material-for-next-Generation-Li-ion.pdf). For IC-type positive electrodes, particle size and surface composition are important in regard to their electrochemical properties. Approaches include the utilisation of sub-micro and nano-sized particles, single crystals and electrolyte-compatible surface coating layers, have been proven to be effective^36,75^.

### Evaluation of energy and power performances at the cell level

Specific energy and energy density are the most important parameters used to assess LIB cells because they consider not only cell capacity and voltage but also the weight and volume of all active/inactive components^76^. In general, the target-specific energy and energy density can be estimated via the top-down approach slicing from the pack level to the electrode and/or material level using in-house developed calculation tools^77^ or by the bottom-up approach starting from theoretical values^78^. Realistic values for the energy density of Si/Si-B/Si-D-based electrodes have to be considered in the lithiated state; hence, the displaced electrolyte volume and single electrode energy density cannot be measured in practice without considering the counter electrode^42,79^. As discussed in the ‘Energy density estimation of full cell’ section, the achievable energy density of Si/Si-B/Si-D||IC cells is associated with several parameters at the material level. To date, substantial efforts have been devoted to the nano-structural design of Si/Si-B/Si-D anodes aiming to improve Li^+^ and *σ*_e−_ diffusivity and reduce volume change^16^. The high specific energy/energy density and rate capability of Si/Si-B/Si-D anodes have been extensively reported in recent years, reaching high areal loadings and capacities (>10 mg cm^−2^ and >10 mAh cm^−2^) and extremely high C-rates (10 C)^80–90^. However, most of the results are obtained from the testing of laboratory-scale cells assembled in electrolyte-flooded conditions (i.e. E/AM > 5) and using a Li-metal electrode as the counter electrode (i.e. half-cell setup with N/P > 3)^11,12,21^.

Considering the ambiguous use of ‘specific energy and energy density’ within the research of Si/Si-B/Si-D||IC cells (i.e. misuse of energy values obtained at different calculation levels), it is necessary to give an explicit definition of this term depending on the technology readiness level (TRL). Figure 6F schematically portrays the evolution of energy density from a low TRL (i.e. materials discovery and testing in laboratory research) to a high TRL (industrial battery pack assembly). At the material level, a number of enticing values have been reported in the literature, such as high specific energy of 700 Wh kg^−1^^80^. However, these values decrease drastically after shifting to the cell level when the mass of the electrolyte, separator, current collectors, and conductive additives are included in the calculation of the specific energy and energy density (see Supporting Fig. 1 for details). In general, various factors can be discounted when translating the Si electrode specific capacity to the practical specific energy and energy density of a Si/Si-B/Si-D||IC cell. Our calculations and those from other groups^84^, indicate that an improvement of >20% in terms of specific energy and energy density can be achieved for Si/Si-B/Si-D||IC cells compared to current Li-ion cell technology.

With very limited information related to the porosity of the electrode and density of each component, it is very difficult to estimate the specific energy and energy density, thereby leading to difficulty in expounding the power/rate capabilities at the cell level for the results reported in the literature. Although recent reports have included key parameters such as the E/AM and N/P ratios, along with the areal capacity^88–91^. It must be stressed that transversal comparison between the specific energy/energy density and rate/power capabilities at the cell level can only be made when all the essential components (Fig. 6F) of the cell are considered for calculating the specific energy and energy density. Herein, we recommend the consideration of the following parameters as compulsory when reporting new results related to Si/Si-B/Si-D||IC cells:

1. Electrode materials: mass percentage and density of the individual components (active materials) within the electrode; porosity, thickness, mass loading and nominal capacity of active material.
2. Electrolyte and separator: density and volume of electrolyte filled for each cell, thickness and density of separator.
3. Other relevant parameters: N/P ratio, details related to current collectors, and cell configurations.

### Safety-induced risks

The large-scale deployment of LIB cells necessitates the appraisal of safety-induced risks, including the heat release rate (HRR), enthalpy of combustion (Δ*H*_c_), and release and dose of toxic products, under various abuse conditions. The legitimate questions associated with Si/Si-B/Si-D||IC cells could be as follows: how do the intrinsic (linked to the properties of the material, such as the easily breakable SEIs of Si/Si-B/Si-D and structural instability of ICs) safety issues of Si/Si-B/Si-D anodes and Ni/Li-rich/high-voltage cathodes affect the safety of Si/Si-B/Si-D||IC full cells; how does the low diffusivity of Li^+^ ions and electrons in a Si/Si-B/Si-D anode, compared to those in Gr electrodes, delay the thermal runaway; and how does the large volume change, electrode swelling and instability of the SEI layer affect the safety issues of practical Si/Si-B/Si-D anode-based full cells? The answers to the above questions and related (intriguing) queries require a thorough understanding of the safety-related patterns of the individual electrodes and full cells, making use of various analytical tools.

### Material to cell cost

The overall cost of Si/Si-B/Si-D||IC originates from the raw sources, synthesis and processing, electrolytes, binders, current collectors, conductive additives, formation stages and other factors. From the anode side, although Si precursors are inexpensive (e.g. SiO_2_), the cost linked to the large-scale production of realistic Si/Si-B/Si-D-containing cells could pose a major challenge. A high abundance of economical starting materials does not necessarily mean that the final product is market competitive. For instance, the large-scale production of nano-Si demands a complex process with sophisticated techniques; therefore, it is pricier than upstream materials such as SiO_*x*_ nano-silica, micro-Si, metallurgical Si wafers and Si from rice husk (negligible price)^24,50,92^. From the cathode perspective, the cost related to Co and Ni in IC materials as well as large-scale manufacturing is a pressing issue^15,93^.

Importantly, in addition to the active materials and associated costs, electrolyte wetting and SEI/CEI formation are among the most expensive steps in battery production ecosystems^94,95^. Because of slow wetting and charge/discharge rates, electrode processing and wetting/formation costs are 2.2 and 7.5 $ kW h^−1^, respectively. Depending on the cell manufacturer, cell chemistry and other factors (e.g. temperature), this process can take 15–21 days, demanding a large amount of floor space as well as large amounts of consumed energy for the cyclers and environmental chambers^96^. The electrode processing and wetting/formation processes, particularly for Si||IC cells, are detrimental features towards the overall cost and need ample attention. Overall, the economic estimation of Si/Si-B/Si-D||IC cells demands a holistic approach as well as a meticulous assessment of the overall cost of LIB production.

## Summary and outlook

**Fig. 7:**
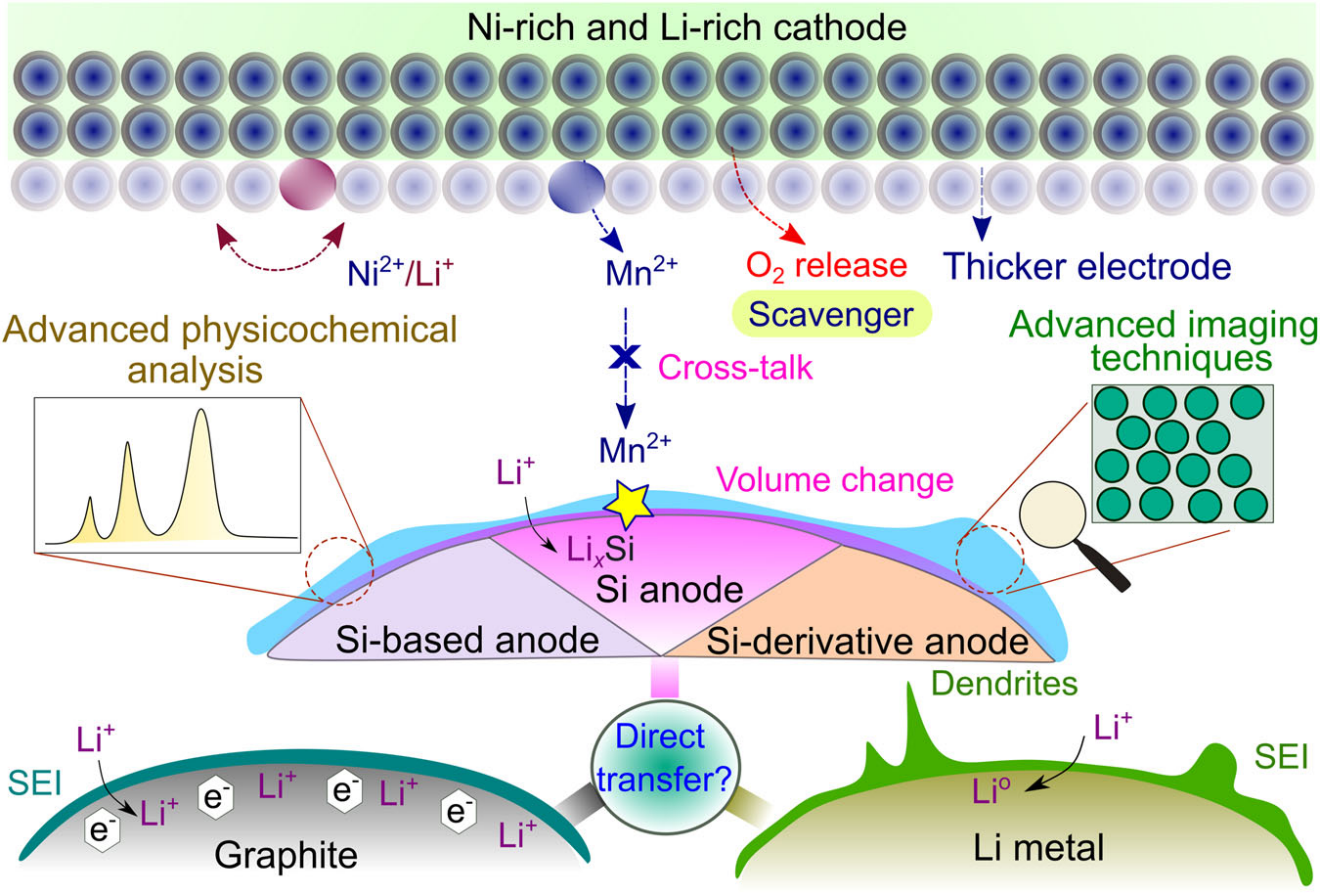
Challenges and solutions for Si/Si-B/Si-D||IC cells. To implement this technology commercially, Si/Si-B/Si-D anodes suffer from a very large volume change, with ICs mostly hindered by their chemical/electrochemical instability (e.g. cation displacement, dissolution of transition metal ions, release of reactive oxygen, etc.). Shifting from the atomic/material level to the cell level, crosstalk between anode and cathode materials during continuous cycling and thick electrodes are required for high-energy batteries, although this may impose additional challenges for stabilising electrode/electrolyte interphases. In addition, the direct transfer of knowledge attained from graphite and Li metal-based rechargeable batteries to Si/Si-B/Si-D||IC systems is not straightforward. Solutions may involve advanced physicochemical analysis and imaging techniques to understand anode-electrolyte interphases as well as scavengers for capturing O_2_ gas.

In consideration of their potential advantages, such as their low cost, environmental benignity, and high energy, batteries built on Si, Si-B and/or Si-D coupled with IC are gaining exceptional momentum. However, combined approaches and solutions with synergistic effects are needed to overcome the obstacles associated with Si/Si-B/Si-D||IC cells. Figure 7 schematically presents the critical factors that need to be contemplated as guidelines along with the solutions for the incorporation of these systems. For the development of practical Si/Si-B/Si-D||IC cells, the following criteria are suggested as future directions to boost the practical performance metrics required by battery manufacturers.

### Material-level design

#### Selection and elaboration of electrode materials

Considering the overall cell performance of Si/Si-B/Si-D||IC cells, Si/Gr with Si ≈ 40 wt.%, SiO/Gr and SiN_*x*_/C blend/composite anodes along with Ni/Li-rich cathode materials could be regarded as materials of choice. Due to the extra embedded Li, Li-rich cathode materials coupled with Si/Si-B/Si-D are also worth investigating. Regarding the anode side, surface coating/modification, surface engineering plus tuning morphologies could be developed. Regarding the cathode side, strategies including examining critical synthesis conditions, electrode modification and regulating the interphases could be further explored.

#### Electrolyte additives and salt anions

The development of molecular and/or salt-type additives with multiple functionalities and/or synergistic effects is important to advance the performance of future Si/Si-B/Si-D||IC cell generations. To speed up the discovery of novel electrolyte additives and salt anions, high-throughput screening methods via combined theoretical, electrochemical, and spectroscopic methods are in great demand.

#### Design of polymeric binders

Binders with abundant –O– or –N– and highly polarised moieties such as –COOH, –OH, and –NH_2_ bestow moderately strong but elastic (reversible/self-healing) supramolecular interactions such as hydrogen bonding, ion-ion interactions, ion-dipole interactions, etc. In addition, in-depth research on structure (bond strength)-property correlations could further elucidate the working mechanisms of these binders in Si/Si-B/Si-D||IC cells.

#### Current collectors

For high areal capacity electrode materials, their efficient implementation requires physical contact between the electrode coatings and current collectors. Replacing flat current collectors with three-dimensional architectures obtained from cost-effective approaches could functionally increase the contact area and promote the transport of electrons within thick electrodes, improving the capacity and cyclability of these high-energy cells. As a result, the dedicated effort is needed for the design and elaboration of current collectors for Si/Si-B/Si-D||IC cells.

### Active material structuring

Building a stable electrode structure is among the most effective approaches to enable the practical application of Si- and Si-based LIBs as well as derivative anode-based LIBs^97–99^. This includes Si nanoparticles (NPs), 3D architectures, aligned nanowires/nanotubes, dispersal of Si into an active matrix, Si-based thin films, and free standing and other tailored morphologies. The engineering of Si nanostructure materials, including 0-dimensional (0D) nanoparticles, 1D nanowires, 2D nanosheets, and 3D hierarchical nanostructures, has been proven to be an effective strategy to reduce the massive volume change of Si and improve the interaction between Si active materials and electrolytes due to the larger surface area^100^. Nano-sized Si particles further promote electrical conductivity by shortening the transport distance for e^−^/Li^+^ and reducing the inhomogeneous lithium diffusion-induced stress and strain. However, nanoparticles also induce accelerated electrolyte decomposition and thickening of the SEI, resulting in high impedance, decreased ICE, and poor thermal stability; all of the above cumulatively limits their future commercial viability^100^. It is noteworthy to mention that the above strategies have only been demonstrated at the laboratory scale.

### Processing of electrode and electrolyte materials

The cell-level processing of both Si/Si-B/Si-D anodes and IC materials plays a key role in designing high-performance electrochemical energy storage devices. These entail optimisation of slurry formation and electrode processing, electrolyte formulation, and the N/P ratio, particularly towards the implementation of thick electrodes, which are critical for achieving sufficient energies. In short, process optimisation, cell testing conditions and protocols at different levels are important to transpose laboratory results into commercial devices.

### Pre-lithiation

Pre-lithiation via the addition of scalable and safe materials acting as lithium-ion donors appears to be the most promising approach to boost the practical energies of Si/Si-B/Si-D||IC cells. This may finally lead to the commercial implementation of such high-capacity anode materials (i.e. Si content >10 wt.%).

### Half-cells to full cells data transfer and reporting

The electrochemical energy storage performance discrepancy between the laboratory-scale half-cells and full cells is remarkable for Si/Si-B/Si-D negative electrodes and IC positive electrodes. Unlike the infinite access of cyclable Li^+^ ions supplied from the metal anode in half-cells, the supply in full cells is limited by the capacity of the cathode. The consumption of cyclable Li^+^ from parasitic (side) reactions results in notable capacity losses in full cells compared to half-cells. Aside from the Li inventory, issues such as electrolyte drying out and optimisation of the N/P ratio also hinder the practicality of full cells. Consequently, accurate and complete data reporting would accelerate the projection of laboratory results into industry-implemented values.

### Lessons from current research: out-of-the-box thinking for the future

Although the inspiration and knowledge gained from the present LIBs can provide insights to improve the understanding of high-energy Si/Si-B/Si-D||IC cells, there is no guarantee that the accumulated knowledge can be transferred. The lack of standard testing protocols for laboratory-based investigations leads to hyperboles; approaches used in one system may not be applicable to evaluate new battery materials and technologies. The technology (in general), the screening of additives, polymeric binders, electrode design/engineering and utilisation of half/full cells calls for out-of-the-box thinking for new methodologies.

### System consideration: anode-cathode crosstalk

The interfacial processes and associated chemistries occurring at the polarised electrodes, electrolyte and formed interphases are usually considered isolated from each other. However, ever-increasing evidence discloses that certain forms of crosstalk exist between them. This is because the species generated on one electrode often appear on the other compartment with unforeseen detrimental effects. This issue becomes critical with Si/Si-B/Si-D||Ni/Li-rich batteries because both electrode compartments are endowed with harsh (electro)chemical conditions (e.g. an unstable SEI layer on the anode and active oxygen release on the cathode). Low potential (e.g. 0.2 V vs. Li/Li^+^) lithiation processes cause an increase in the predetermined voltage of the cathode upon repeated cycling, resulting in decreasing cycling efficiencies, promoting capacity fade and increasing impedance. Hence, the design of high-energy Si/Si-B/Si-D||IC cells entails a systemic and detailed study of the crosstalk between electrodes, electrolytes and interphases along with their complex chemistries.

### Advanced physicochemical analysis and imaging techniques

Despite the vast interest in the coupling of high-capacity Si/Si-B/Si-D and IC electrodes, a detailed understanding of the underlying active/inactive mechanisms under practical conditions remains unknown. Despite the synergistic positive effect due to coupling, understanding such processes requires advanced physicochemical analytical tools. In consideration of the harsh conditions on both compartments and their possible crosstalk, an in-depth fundamental study using real-time in situ experimental techniques coupled with theoretical calculations is necessary. Making use of sub-molecular and atomic-level simulations assisted by the further discovery of optimisation strategies is of paramount importance to transition towards their commercialisation. The failure mechanisms deriving from changes in the chemical composition, morphology and crystal structure of electrochemically active components still require a basic understanding.

Finally, the authors would like to propose the following open-ended questions to the scientific community to help improve understanding and to tackle some of the challenges linked to Si-containing/derivative anodes and IC cathodes.

1. Considering the high operating potential and massive volume change associated with Si, what will be the real contribution of Si and SiO in Si-Gr and SiO-Gr blends/composites, respectively, towards the practically obtainable energy density in terms of the driving range of EVs?
2. How does the large volume change of Si-based electrodes affect the volumetric energy of the full cells? How does the cathode respond to the large volume change of Si?
3. How does the fraction of incoming Li^+^ from pre-lithiated Si-Gr, and Si/SiO blend/composite electrodes compare for Gr, Si and SiO, in relation to an identical non-pre-lithiated cell?
4. What are the governing failure mechanisms of Si-Gr and SiO/Gr blend/composite electrodes from short- and long-term cycling perspectives, particularly in full cell configurations?
5. How does the amount of nitrogen and/or Si affect the capacity, cyclability, chemical and electrochemical stability of the conversion and alloying reactions?
6. How does the cation migration and active oxygen release from ICs affect the volume change associated with Si, Si-based and Si-derivative materials in full cell configurations?
7. How does a single crystal IC-type positive electrode affect the overall properties of a full cell?

In conclusion, high-energy-density Si/Si-B/Si-D||IC cells have received significant attention from both the academic and industrial research communities. With extensive innovative R&D, Si/Si-B/Si-D||IC cells could be deemed as future generation technologies for use in emerging large-scale applications, such as their use in EVs and for the efficient integration of renewable energy sources.

## Supplementary information

Supplementary Information
