# Supplementary material for: Production of high-energy Li-ion batteries comprising silicon-containing anodes and insertion-type cathodes
Source: Nat Commun. 2021 Sep 15;12:5459. doi: 10.1038/s41467-021-25334-8 (PMC8443554; doi:10.1038/s41467-021-25334-8)
Supplement: Supplementary file 1 — Supplementary Information [file 41467_2021_25334_MOESM1_ESM.docx]

**Supplementary Information for**

**Production of high-energy Li-ion batteries comprising silicon-containing anodes and insertion-type cathodes**

Gebrekidan Gebresilassie Eshetu,^1,2,†^ Heng Zhang,^3, †^ Xabier Judez,^4^ Henry Adenusi,^5,6,7,8^ Michel Armand,^4^ Stefano Passerini,^5,6,8^* and Egbert Figgemeier^1,9,^*

^1^ Institute of Power Electronics and Electric Drives, ISEA, RWTH Aachen, Jägerstraße 17/19, 52066, Aachen, Germany

^2^ Department of Material Science and Engineering, Mekelle Institute of Technology—Mekelle University, Mekelle 1632, Tigray, Ethiopia

^3^ Key Laboratory of Material Chemistry for Energy Conversion and Storage (Ministry of Education), School of Chemistry and Chemical Engineering, Huazhong University of Science and Technology, Luoyu Road 1037, 430074, Wuhan, China

^4^ Centre for Cooperative Research on Alternative Energies (CIC energiGUNE), Basque Research and Technology Alliance (BRTA), Alava Technology Park, Albert Einstein 48, 01510 Vitoria-Gasteiz, Spain

^5^ Karlsruhe Institute of Technology (KIT), P.O. Box 3640, 76021, Karlsruhe, Germany

^6^ Helmholtz Institute Ulm (HIU), Helmholtzstraße 11, 89081, Ulm, Germany

^7^ Hong Kong Quantum AI Lab (HKQAI), 17 Science Park West Avenue, Hong Kong Science Park, Pak Shek Kok, New Territories, Hong Kong, China

^8^ Department of Chemistry University of Rome “La Sapienza”, Piazzale Aldo Moro 5, 00185 Rome, Italy

^9^ Helmholtz Institute Münster (HI MS), IEK-12, Forschungszentrum Jülich, Corrensstrasse 46, 48149, Münster, Germany

**Corresponding Authors:* stefano.passerini@kit.edu; e.figgemeier@fz-juelich.de

^†^These authors contributed equally to the work.

**Supporting Table 1**. Characteristics (ca. density of pristine, reaction potential, density of lithiated phase, theoretical gravimetric and volumetric capacities, volume variation upon lithiation, Li diffusion, level of commercialisation etc.) of selected anode materials (i.e.; graphite, LTO, Li, Si and SiO*_x_*).^1,2^

| Anode Material | C | Li_4_Ti_5_O_12_ | Li | Si | SiO*_x_* |
| --- | --- | --- | --- | --- | --- |
| Density / g cm^−3^ | 2.25 | 3.5 | 0.53 | 2.3 | 2.13 |
| Lithiated Phase | LiC_6_ | Li_7_Ti_5_O_12_ | Li | Li_3.75_Si^[a]^  Li_4.4_Si^[b]^ | SiO^[e]^  *x* ~1 |
| Reaction Potential vs. Li/Li^+^ / V | 0.05 | 1.55 | 0 | 0.31 (*x* = 3.75)  0.25 (*x* ~4.4) |  |
| Density of lithiated phase / g cm^−3^ | 2.20 | 3.65 | 0.53 | 1.18 |  |
| Theoretical Capacity / mAh g^−1^ | 372 | 175 | 3862 | 3590^[a]^  4200^[b]^ | 1710 |
| Theoretical volumetric capacity / mAh cm^−3^ | 837 | 613 | 2061 | 8360^[a]^  9660^[b]^ | 3172 |
| Volume variation / % | 10–12 | 1 | 100 | > 280 | 160 |
| lithium diffusion coefficient / cm^2^ S^−1^ | 10^−11^–10^−7^ | 10^−12^–10^−11^ |  | 10^−13^–10^−11^ |  |
| Commercialisation | Y | Y | Y^[c]^ | Y^[d]^ | Y^[e]^ |
| ^[a]^ @ room temperature, ^[b]^ @ higher temperature (415 ^o^C), ^[c]^ By Bolloré, ^[d]^ As dopant for graphite (Si-Gr composite, < 10 wt%), ^[e]^ As dopant for graphite (SiO*_x_*-Gr composite, < 10 wt.%), Y: yes | | | | | |

**Supporting Table 2**. Discharge capacity (*C*_NMC_), thermal stability [peak temperature (*T*_peak_) and heat generation (∆*H*)], electronic conductivity (*σ*_e_) and capacity retention (CR) of NMC materials with various Ni contents.^3^

| Composition ^[a]^ | *C*_NMC_ / mAh g^−1^ | *T*_peak_ / ^o^C | ∆*H* / J g^−1^ | *σ*_e_ / S cm^−1^ | CR / % |
| --- | --- | --- | --- | --- | --- |
| 0.33 | 163 | 306 | 512.5 | 5.2E-8 | 92.4 |
| 0.5 | 175 | 290 | 605.7 | 4.9E-7 | 90.0 |
| 0.6 | 187 | 264 | 721.4 | 1.6E-6 | 85.1 |
| 0.7 | 194 | 242 | 826.3 | 9.3E-6 | 78.5 |
| 0.8 | 203 | 232 | 904.8 | 1.7E-5 | 70.2 |
| 0.85 | 206 | 225 | 971.5 | 2.8E-5 | 66.4 |
| ^[a]^ Molar ratio of Ni in NMC cathodes. | | | | | |

Realistic calculations of gravimetric and volumetric energies at cell level were performed on the basis of a previously described model.^1,2^ In principle, the energy of battery cells could be calculated by dividing total cell energy (i.e., product of cell capacity and potential difference between cathode and anode) by either the total mass (gravimetric energy, *E*^g^_cell_, **Supporting Equation 1**) or volume of the whole cell components (volumetric energy, *E*^v^_cell_, **Supporting Equation 2**).

$E_{\mathrm{cell}}^{g}=\frac{C_{\mathrm{cell}} \times V_{\mathrm{cell}}}{m_{curr. col.}+m_{\mathrm{cathode}}+m_{\mathrm{anode}}+m_{\mathrm{electrolyte}}+m_{\mathrm{anode}}}$ (**Supporting Equation 1)**

$E_{\mathrm{cell}}^{v}=\frac{C_{\mathrm{cell}} \times V_{\mathrm{cell}}}{v_{curr. col.}+v_{\mathrm{cathode}}+v_{\mathrm{anode}}+v_{\mathrm{electrolyte}}+v_{\mathrm{anode}}}$  **(Supporting Equation 2)**

In **Supporting Equations 1** and **2**, *m*, *v*, C, V, *E^g^* and *E^v^* represent mass, volume, capacity, cell voltage, gravimetric and volumetric energies, respectively. The parameters used for the calculations are summarised in **Supporting Tables 3** and **4**, and further details can be found in the literature.^4,5^

**Supporting Table 3.** The properties of various active materials used for the calculations.

|  | Capacity / mAh g^−1^ | Discharge voltage / V vs. Liº/Li^+^ | Density / g cm^−3^ |
| --- | --- | --- | --- |
| Lithium | 3861 | 0.00 | 0.53 |
| Silicon | 3590 | 0.10 | 2.33 |
| Graphite | 372 | 0.10 | 2.20 |
| LiNi_0.8_Co_0.15_Al_0.05_O_2_ (NCA) | 220 | 3.70 | 4.60 |
| LiNi_0.33_Mn_0.33_Co_0.33_O_2_ (NMC111) | 160 | 3.70 | 4.75 |
| LiNi_0.5_Mn_0.3_Co_0.2_O_2_ (NMC532) | 180 | 3.70 | 4.65 |
| LiNi_0.8_Mn_0.1_Co_0.1_O_2_ (NMC811) | 220 | 3.70 | 4.65 |
| LiCoO_2_ (LCO) | 140 | 3.80 | 5.05 |
| LiNi_0.5_Mn_1.5_O_4_ (LNMO) | 135 | 4.70 | 4.40 |

**Supporting Table 4.** Characteristic of anodes, insertion type cathodes and electrolyte materials employed for the calculations.

| Parameters | Anode | Cathode^[a]^ |
| --- | --- | --- |
| Current collector thickness / µm | 4 | 10 |
| Current collector weight / mg cm^–2^ | 3.58 | 2.70 |
| Active material / vl% | 65 | 65 |
| Binder / vl%^[a]^ | 30/30/30/2 | 30/30/30/2 |
| Binder density / g cm^–3^ | 1.20/1.95/5.15/1.76 | 1.20/1.95/5.15/1.76 |
| Free electrolyte / vl% | 0/0/0/28 | 0/0/0/28 |
| Free electrolyte density / g cm^–3^ | 0/0/0/1.13 | 0/0/0/1.13 |
| Carbon / vl% | 5 | 5 |
| Carbon density / g cm^–3^ | 2.20 | 2.20 |
| Porosity / % | 20/10/10/10 | 20/10/10/10 |
| Areal capacity ratio of negative/positive electrode | 1.05 | N.A. |

^[a]^ The values separated by slash refer to those for polymer/glassy/ceramic/liquid electrolytes, respectively.





**Supporting Figure 1. Comparison between material and cell level energies.**

(A, B) Specific energy (A) and energy density (B) vs. Si fraction for LE-based Si/Gr || NMC811 cells.

(C, D) Loss ratio in specific energy (C) and energy density (D) for LE-based Si/Gr || NMC811 cells.

As seen from **Supporting Figure 1**, at material level, the Si/Gr || NMC811 cells holds quite high *E*_g_ and *E*_v_ values, particularly, at high Si fractions (e.g., > 700 Wh kg^−1^ and > 2500 Wh L^−1^ for the cell containing > 40% Si). The introduction of inactive materials (e.g., conductive carbon, binder, current collectors, electrolyte, etc.) cause a notable decline in achievable energies at cell level. For example, at a fixed areal capacity of 3 mAh cm^−2^, the loss ratios in *E*_g_ and *E*_v_ are around 45% and 60%, respectively. Interestingly a higher areal capacity minimises the loss in both *E*_g_ and *E*_v_ (e.g., ca. 25% and 50% loss ratios in *E*_g_ and *E*_v_ for the cell with a fixed areal capacity of 10 mAh cm^−2^), which further emphasises the importance of thick electrode in achieving sufficient energies for Si/Si-B/Si-D || IC battery systems.

**Supplementary References**

1 Obrovac, M. N. & Chevrier, V. L. Alloy negative electrodes for Li-ion batteries. *Chem. Rev.* **114**, 11444-11502 (2014).

2 Liu, D. *et al.* Group IVA Element (Si, Ge, Sn)-based alloying/dealloying anodes as negative electrodes for full-cell lithium-ion batteries. *Small* **13**, 1702000 (2017).

3 Bak, S.-M. *et al.* Structural changes and thermal stability of charged LiNi*_x_*Mn*_y_*Co*_z_*O_2_ cathode materials studied by combined in situ time-resolved XRD and mass spectroscopy. *ACS Appl. Mater. Inter.* **6**, 22594-22601 (2014).

4 Judez, X. *et al.* Opportunities for rechargeable solid-state batteries based on Li-intercalation cathodes. *Joule* **2**, 2208-2224 (2018).

5 Li, C. *et al.* Estimation of energy density of Li-S batteries with liquid and solid electrolytes. *J. Power Sources* **326**, 1-5 (2016).
